# The Population Problem

**Published:** 1915-12-18

**Authors:** Binnie Dunlop

**Affiliations:** 24 Alexandra Court, S.W.


*To the Editor of* The Hospital.

### "The Population Problem."

*To the Editor of* The Hospital.

Sir,—As many of your readers seem to be under a misapprehension with regard to my letter last week, please allow me to say that my offer was a free one. Indeed, I shall gladly send literature on the population question and birth control on receipt of a post-card containing name and address.

Yours sincerely,

Binnie Dunlop, M.B., Ch.B.

24 Alexandra Court, S.W.

December 15, 1915.

